# Sleep-related breathing disorder in a Japanese occupational population and its association with exercise-induced blood pressure elevation

**DOI:** 10.1038/s41440-024-02050-6

**Published:** 2024-12-05

**Authors:** Minako Inoue, Satoko Sakata, Hisatomi Arima, Ikumi Yamato, Emi Oishi, Ai Ibaraki, Takanari Kitazono, Kenichi Goto

**Affiliations:** 1 Department of Medicine and Clinical Science, Graduate School of Medical Sciences, Kyushu University, Fukuoka, Japan; 2 Center for Cohort Studies, Graduate School of Medical Sciences, Kyushu University, Fukuoka, Japan; 3 Department of Epidemiology and Public Health, Graduate School of Medical Sciences, Kyushu University, Fukuoka, Japan; 4 Department of Preventive Medicine and Public Health, Fukuoka University, Fukuoka, Japan; 5 Department of Health Sciences, Graduate School of Medical Sciences, Kyushu University, Fukuoka, Japan

**Keywords:** Sleep-related breathing disorder, Exercise-induced hypertension, Sympathetic hyperactivity

## Abstract

Sleep-related breathing disorder (SRBD) and exercise-induced blood pressure (BP) elevation are known risk factors for hypertension. However, the relation between them remains unknown. This cross-sectional study examined the relationship between SRBD and exercise-induced BP elevation in a Japanese occupational population. Using the 3% oxygen desaturation index (3%ODI) obtained by a portable monitor for overnight saturation of percutaneous oxygen (SpO2), participants were classified into low (0 ≤ 3%ODI < 5), medium (5 ≤ 3%ODI < 15), and high (15 ≤ 3%ODI) 3%ODI groups. We included employees who had undergone an exercise electrocardiogram test after monitoring for overnight SpO2. In total, 928 employees were included. The median age of the participants was 50 years, 96% were male, the mean body mass index was 23.9 ± 3.1 kg/m^2^, and the median 3%ODI was 4.9 (interquartile range: 1.6–6.5). Among them, 30% and 5% were categorized into the medium and high 3%ODI groups, respectively. At a median exercise intensity of 10.1 METs, BP changed from 124 ± 16/76 ± 12 mmHg before to 183 ± 26/85 ± 14 mmHg after exercise, with a mean systolic BP change of +59 ± 23 mmHg (−20 to +128 mmHg). When we defined systolic BP change of +60 mmHg or more as exercise-induced BP elevation, the odds ratio for exercise-induced BP elevation increased significantly with higher 3%ODI levels after multivariate adjustment for parameters including current use of antihypertensive medication and maximal exercise intensity (p for trend = 0.01). Higher 3%ODI was significantly associated with higher prevalence of exercise-induced BP elevation, suggesting sympathetic hyperactivity occurs in SRBD patients. Our results suggest the potential presence of SRBD should be considered in individuals with exercise-induced BP elevation.

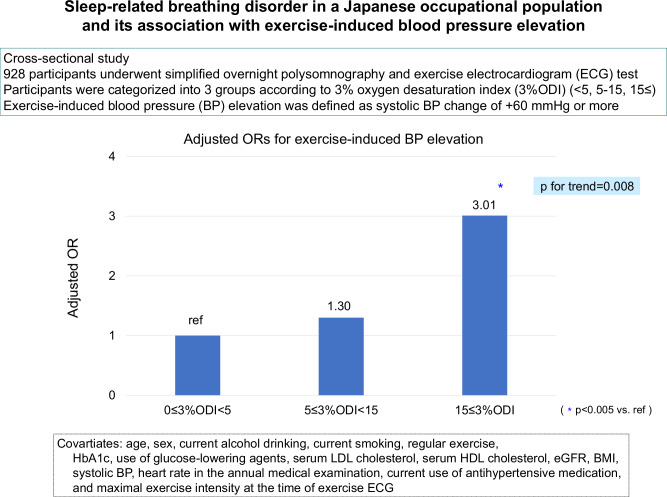

## Introduction

Sleep-related breathing disorder (SRBD) causes hypertension [1], and it is a risk factor for the development of cardiovascular diseases [2–4].

Past epidemiological studies suggest that obstructive sleep apnea (OSA) with an apnea-hypopnea index (AHI) of 5 or more accounts for most cases of SRBD. The AHI represents the total number of apnea and hypopnea incidents per hour during sleep. OSA is one of the causes of secondary hypertension, and it is known that the severity of sleep apnea has a linear relationship with the risk for the development of hypertension [5]. We previously reported that SRBD was associated with hypertension in Japanese occupational population including non-hypertensive participants [6].

Exercise-induced blood pressure (BP) elevation is also reported to be a risk factor for the development of hypertension and okcardiovascular diseases [7–9]. The Framingham study reported that in the general population, increased systolic BP after exercise was associated with an increased risk for the development of hypertension [7]. One of the possible mechanisms may be sympathetic hyperactivity; thus, SRBD could be associated with exercise-induced BP elevation. However, very few studies have examined the association between SRBD and exercise-induced BP elevation.

In this study, we investigated the association between SRBD and exercise-induced BP elevation in a Japanese occupational population.

Point of view

- **Clinical relevance**
  The presence of SRBD should be considered in individuals with exercise-induced systolic BP elevation.
- **Future direction**
  A prospective study to investigate the causal relationship between 3%ODI elevation and exercise-induced BP elevation is warranted.
- **Consideration for the Asian population**
  Asians tend to have small jaws and higher abdominal adiposity, which may strengthen the association between 3%ODI and exercise-induced BP elevation in Asian population.

## Methods

### Study population

This cross-sectional study enrolled employees of a railroad and bus company based in Fukuoka, Japan. Study participants were enrolled between 2012 and 2018, and underwent both overnight monitoring for saturation of percutaneous oxygen (SpO2) using a portable device and an annual medical examination within a year of enrollment. At the railroad and bus company employing the study participants, portable-device monitoring for overnight SpO2 was conducted once every three years for active train and bus drivers who were judged fit for driving with passengers. Of those, 105 drivers who were already on continuous positive airway pressure (CPAP) treatment were excluded. Among the remaining participants, exercise electrocardiogram (ECG) tests were performed on 986 participants from 2014 to 2018. At the company, the exercise ECG tests were conducted once every five years for active train and bus drivers over 40 years of age as a screening for myocardial ischemia. Individuals with documented coronary artery disease who had undergone myocardial ischemia evaluation within the past two years and those with hypertrophic cardiomyopathy were excluded. Twenty-eight participants who performed the exercise ECG tests before the portable-device monitoring for overnight SpO2 and 30 participants whose BP values during exercise were missing were excluded. Finally, 928 participants were enrolled in the present study. When the participants underwent the portable device monitor for overnight SpO2 more than once during the study period, the first set of results was used in this analysis.

The research proposal for this study was prepared in accordance with the “Ethical Guidelines for Medical and Health Research Involving Human Subjects” issued jointly by the Ministry of Education, Culture, Sports, Science and Technology (MEXT) and the Ministry of Health, Labour and Welfare (MHLW) of Japan. Study approval was obtained from the Ethical Review Committee of the Kyushu University Medical School and Hospital. We explained the use of the data for this study at the time of the annual medical examination and posted the content and purpose of this study on the website of the Department of Medicine and Clinical Science, Graduate School of Medical Sciences, Kyushu University. Employees were excluded if they declined the use of their data for this analysis.

### Measurement of BP levels and confounding variables

Physical measurements, administration of lifestyle questionnaires, and blood and urine tests were conducted at the annual health examination. Height, weight, sitting BP, and pulse were measured, and body mass index (BMI) was calculated as weight (kg)/height (m) squared. Obesity was defined as BMI greater than 25 kg/m^2^ [10]. BP values in the sitting position were measured by a mercury sphygmomanometer in 2012 and 2013, and by an electronic oscillometric sphygmomanometer (ES-H55, Terumo Inc., Tokyo) from 2014 to 2018. The measurements were repeated and the steady values of BP were used for the present analysis. Hypertension was defined as BP greater than 140/90 mmHg and/or current use of antihypertensive medication [11]. Heart rate was measured by resting ECG. Blood sampling was performed without consideration for the timing of meals, although, the frequency of fasting blood sampling was higher. Diabetes mellitus was defined as hemoglobin A1c (HbA1c) greater than 6.5% and/or current use of glucose-lowering agents (i.e., oral glucose-lowering agents or insulin) [12]. Blood glucose levels were not used for the definition of diabetes mellitus. Dyslipidemia was defined as serum low-density lipoprotein (LDL) cholesterol greater than 140 mg/dL and/or serum high-density lipoprotein (HDL) cholesterol less than 40 mg/dL and/or current use of lipid-modifying agents [13]. Triglycerides were not used for the definition of dyslipidemia. Estimated glomerular filtration rate (eGFR) was calculated using the chronic kidney disease epidemiology collaboration equation with a Japanese coefficient of 0.813 [14]. Regarding lifestyle, the participants were asked to fill out questionnaires on drinking, smoking, and exercise in advance, and the nurses additionally asked about any missing information or details on the day of the medical checkup. Drinking was defined as drinking at least once a week, regardless of the amount of alcohol consumed. Smoking was defined as current smoking, regardless of the number of cigarettes smoked. The number of cigarettes smoked was expressed as the Brinkman index (number of cigarettes smoked per day multiplied by number of years smoked), regardless of whether or not the individual was currently smoking. Regular exercise was defined as exercising at least twice per week.

### Assessment of sleep-related breathing disorder

In this study, SRBD was assessed using a portable monitor for overnight SpO2 rather than full polysomnography. The portable monitor calculates the 3%ODI, which is the number of the times when SpO2 decreases by 3% or more per hour. 3%ODI is well known to correlate with AHI and is often used as a screening test. The validity of the portable monitor for overnight SpO2 has been confirmed in several studies [15–18]. Either of two portable monitor devices was used. The PMP-200GpluxX (Royal Phillips Co., Amsterdam, Netherlands) or PULSOX-Me300 (Minolta Co., Osaka, Japan). Participants performed the test at home, as instructed by nurses. After attaching a pad to the wrist of the non-dominant arm, the sensor probe was attached to the index finger and secured with the provided tape. The power button was held down for at least 2 s, and the monitor on the pad confirmed that the measurement had started. The collected data were analyzed by Philips. SRBD was defined according to the International Classification of Sleep Disorder, 3rd Edition (ICSD-3), with 3%ODI 5 events/h or more considered mild, and 3%ODI ≥ 15 events/h considered moderate or severe [19].

### Assessment of BP change during exercise ECG

Exercise ECG tests were performed by cardiologists at seven medical facilities commissioned by the railroad and bus company. A brief interview by a cardiologist was performed prior to testing. No participants had cardiovascular contraindications to the exercise testing [20]. The Bruce protocol was used for the treadmill method [21]. Individuals who were presumed to have high exercise capacity based on a pre-test interview were allowed to shorten the low-intensity stage at the discretion of the cardiologists. The endpoints were the appearance of new symptoms, new significant findings on the ECG, exercise-induced BP abnormalities (when systolic increased BP ≥ 250 mmHg or decreased by 10 mmHg), or reaching the target heart rate (THR) [22, 23]. The THR was calculated as (220-age) × 0.85. Baseline and maximum heart rate, BP, duration of exercise, and workload achieved (maximal exercise intensity) were recorded. BP at baseline was measured in the supine position.

### Statistical analysis

The distributions of variables were evaluated by visually checking the histograms and by performing normality test using Kolmogorov-Smirnov test and Anderson-Darling test. Consequently, the distributions of age, the Brinkman index, maximum exercise intensity, and exercise duration were skewed. The trends in the means (standard deviations) and the frequencies of risk factors across the levels of 3%ODI were estimated by a linear and a logistic regression analysis, respectively. The trends in the medians (interquartile ranges) across the levels of 3%ODI were estimated by a nonparametric method (Wilcoxon rank sum test).

Previous studies have estimated an average BP increase of +60 mmHg in men and +50 mmHg in women during exercise [7.23]. In addition, a previous case-control study defined a BP increase of +60 mmHg in men and +50 mmHg in women as an exaggerated BP response to exercise and reported that significantly greater neurohormonal responses were observed in the individuals with an exaggerated BP response to exercise than in the individuals with normal BP reactivity [24]. Following these previous findings, we defined systolic BP change of +60 mmHg or more as an exercise-induced systolic BP elevation. In a sensitivity analysis, participants were divided into quartiles based on the degree of systolic BP change, and the highest quartile was defined as exercise-induced systolic BP elevation. Adjusted BPs (standard errors) and adjusted odds ratios (95% confidence intervals) for exercise-induced BP elevation according to 3%ODI levels were estimated by using analysis of covariance and logistic regression models, respectively. All analyses were conducted using an age- and sex-adjusted model as well as a multivariate adjustment model with adjustment for age, sex, current alcohol drinking, current smoking, regular exercise, HbA1c, use of glucose-lowering agents, serum LDL cholesterol, serum HDL cholesterol, eGFR, BMI, systolic BP, heart rate at the annual medical examination, current use of antihypertensive medication, and maximal exercise intensity at the time of exercise ECG. These factors were chosen both because the specific backgrounds of our participants suggested they would be potential confounding factors and because they have been adopted as adjustment factors in similar previous studies [25, 26]. All statistical analyses were performed using the SAS program package version 9.4 (SAS Institute, Cary, NC). *P* values < 0.05 were considered significant.

## Results

The median age of the participants was 49 years (interquartile range: 46–54 years), 96.2% were male (894 males and 34 females), and 15.2% (141 participants) were taking antihypertensive medication at the time of portable-device monitoring for overnight SpO2.

The median 3%ODI was 3.3 events/h (interquartile range: 1.6–6.5). The frequencies of mild SRBD (5 ≤ 3%ODI < 15) and moderate or higher SRBD (3%ODI 15 or higher) were 30% and 5%, respectively. Table 1 shows the baseline characteristics of the total study population and the mean values or frequencies of potential risk factors for exercise-induced BP elevation according to the 3%ODI levels. The mean values of BMI, systolic and diastolic BP, and heart rate. HbA1c, serum total cholesterol, serum LDL cholesterol, and serum HDL cholesterol increased significantly with higher 3%ODI levels. The mean values of eGFR decreased significantly with higher 3%ODI levels. The frequencies of obesity, current alcohol intake, lipid-modifying agent use, dyslipidemia, antihypertensive medication, and hypertension increased significantly with higher 3%ODI levels.

**Table 1:** Baseline characteristics of the total study population

		3% oxygen desaturation index			
	All participants	0 ≤ 3%ODI < 5	5 ≤ 3%ODI < 15	15 ≤ 3%ODI	*p* for trend
	(*n* = 928)	(*n* = 608)	(*n* = 279)	(*n* = 41)	
Age (years)	50 (46–54)	49 (46–54)	50 (46–54)	51 (46–54)	0.09
Sex (men, %)	96.3	95.7	97.5	95.5	0.37
Body mass index (kg/m^2^)	23.9 ± 3.1	23.2 ± 2.9	24.9 ± 2.9	26.3 ± 3.8	<0.0001
Obesity (BMI ≥ 25 kg/m2, %)	32.2	23.7	46.7	61.4	<0.0001
Current alcohol intake (%)	62.0	59.8	65.7	72.7	0.08
Current smoking (%)	47.5	48.0	47.8	27.3	0.03
Brinkman index	460 (140–620)	450 (120–600)	480 (180–660)	500 (130–720)	0.18
Regular exercise (%)	15.1	16.0	14.2	15.9	0.78
Systolic BP (mmHg)	122 ± 12	121 ± 11	124 ± 13	124 ± 9	<0.0001
Diastolic BP (mmHg)	79 ± 8	78 ± 8	81 ± 9	82 ± 9	<0.0001
Heart rate (bpm)	72 ± 11	72 ± 12	72 ± 11	75 ± 1	<0.0001
Antihypertensive medication (%)	15.2	9.6	19.0	27.3	0.0004
Hypertension (%)	24.9	16.8	31.8	38.6	0.0002
HbA1c (%)	5.8 ± 0.6	5.7 ± 0.5	5.8 ± 0.6	5.7 ± 0.4	<0.0001
Glucose-lowering agent use (%)	2.5	1.5	2.8	0.0	0.98
Diabetes Mellitus (%)	7.1	4.0	8.3	4.6	0.59
Serum total cholesterol (mg/dL)	213 ± 33	212 ± 32	215 ± 36	220 ± 39	<0.0001
Serum LDL cholesterol (mg/dL)	131 ± 30	130 ± 30	133 ± 31	135 ± 33	<0.0001
Serum HDL cholesterol (mg/dL)	57 ± 16	59 ± 17	54 ± 12	55 ± 14	<0.0001
Lipid-modifying agent use (%)	9.6	6.3	11.4	11.4	0.43
Dyslipidemia (%)	48.8	42.6	54.3	52.3	0.13
eGFR (mi/min/1.73m^2^)	77 ± 11	77 ± 11	76 ± 11	77 ± 12	<0.0001

Data are presented as the mean values (standard deviation), percentages, or median (interquartile range) 3% *ODI* 3% oxygen desaturation index, *BMI* body mass index, *BP* blood pressure, *HbA1c* Hemoglobin A1c, *LDL* Low-Density Lipoprotein, *HDL* High-Density Lipoprotein, *eGFR* estimated Glomerular Filtration Rate

Overall, at a median exercise intensity of 10.1 METs (interquartile range: 7.0–10.2 METs), BP changed from 124 ± 16/76 ± 12 mmHg before exercise to 183 ± 26/85 ± 14 mmHg after exercise, with a mean systolic BP change of +59 ± 23 mmHg (−20 mmHg at minimum to +128 mmHg at maximum). When we investigated the difference of systolic BP change during exercise according to 3%ODI levels, systolic BP was significantly elevated with higher 3%ODI levels (*p* for trend < 0.0001). When we defined systolic BP change of +60 mmHg or more as exercise-induced systolic BP elevation, the frequencies of exercise-induced systolic BP elevation were significantly greater with higher 3%ODI levels (Table 2).

**Table 2:** Results of exercise ECG and echocardiography according to 3%ODI levels in all subjects

		3% oxygen desaturation index			
	All participants	0 ≤ 3%ODI < 5	5 ≤ 3%ODI < 15	15 ≤ 3%ODI	*p* for trend
	(*n* = 928)	(*n* = 608)	(*n* = 279)	(*n* = 41)	
Systolic BP after exercise (mmHg)	183 ± 26	182 ± 26	186 ± 27	193 ± 28	<0.0001
Diastolic BP after exercise (mmHg)	85 ± 14	84 ± 14	85 ± 13	86 ± 18	<0.0001
Systolic BP change during exercise (mmHg)	59 ± 23	58 ± 22	61 ± 24	67 ± 23	<0.0001
Diastolic BP change during exercise (mmHg)	9 ± 13	9 ± 13	9 ± 12	10 ± 11	<0.0001
Excess systolic BP elevation (%)	49.3	46.4	54.2	65.9	0.01
Maximum exercise intensity (METs)	9.3 (7.0–10.2)	10.1 (7.0–10.2)	10.1 (7.0–10.2)	8.8 (7.0–10.2)	0.045
reaching THR (%)	74.1	73.3	75.2	70.5	0.74
Exercise duration (sec)	405 (301–525)	419 (307–v538)	400 (300–525)	360 (221–423)	0.007

Data are presented as the mean values (standard deviation), percentages, or median (interquartile range) 3% *ODI* 3% oxygen desaturation index, *BP* blood pressure, *THR* target heart rate

Table 3 shows the adjusted systolic BP values and the adjusted odds ratios for exercise-induced systolic BP elevation according to the 3%ODI levels. The age- and sex-adjusted systolic BP values and odds ratios for exercise-induced systolic BP elevation increased significantly with higher 3%ODI levels (*p* for trend < 0.0001). These associations remained significant after adjustment for age, sex, current alcohol drinking, current smoking, regular exercise, HbA1c, use of glucose-lowering agents, serum LDL cholesterol, serum HDL cholesterol, eGFR, BMI, systolic BP, heart rate in the annual medical examination, current use of antihypertensive medication, and maximal exercise intensity at the time of exercise ECG (*p* for trend = 0.01). Moreover, the results remained the same when the Brinkman index was adjusted rather than current smoking (data not shown). On the other hand, diastolic BP change during exercise and 3%ODI levels were not significantly associated.

**Table 3:** Adjusted exercise-induced BP change and the odds ratios for exercise-induced systolic BP elevation according to 3%ODI levels

	Number of subjects	Age- and sex-adjusted	Multivariate adjusted
Exercise-induced systolic BP change			
0 ≤ 3%ODI < 5	608	58.1 ± 8.2	57.8 ± 19.3
5 ≤ 3%ODI < 15	279	61.0 ± 8.4	59.7 ± 19.5
15 ≤ 3%ODI	41	67.4 ± 9.0	68.1 ± 20.0
		*p* for trend < 0.0001	*p* for trend = 0.01
Exercise-induced diastolic BP change			
0 ≤ 3%ODI < 5	608	8.6 ± 5.0	8.4 ± 12.1
5 ≤ 3%ODI < 15	279	8.7 ± 5.1	8.8 ± 12.2
15 ≤ 3%ODI	41	10.2 ± 5.5	10.9 ± 12.5
		*p* for trend < 0.01	*p* for trend = 0.61
	Events/total (%)	Age- and sex-adjusted	Multivariate adjusted
Odds ratio for exercise-induced systolic BP elevation			
0 ≤ 3%ODI < 5	280/608 (46.1%)	1.00 (Reference)	1.00 (Reference)
5 ≤ 3%ODI < 15	150/279 (53.8%)	1.35 (1.01–1.79)	1.30 (0.95–1.78)
15 ≤ 3%ODI	27/41 (65.9%)	2.28 (1.17–4.47)	3.01 (1.43–6.33)
		*p* for trend = 0.01	*p* for trend = 0.008

Data are presented as the adjusted mean values (standard error) or odds ratio (95% CI)Age, sex, current alcohol drinking, current smoking, regular exercise, HbA1c, use of glucose-lowering agents, serum LDL cholesterol, serum HDL cholesterol, eGFR, BMI, systolic BP, heart rate in the annual medical examination, taking antihypertensive medication, and maximal exercise intensity at the time of exercise ECG were adjusted in multivariate analysis

3%*ODI* 3% oxygen desaturation index, *BMI* body mass index, 95%*CI* 95% confidence interval

In a sensitivity analysis, when participants were divided into four groups based on the cutoff value of 3%ODI (3%ODI < 5, 5 to 10, 10 to 15, and 15 or more), similarly significant associations were observed (*p* for trend < 0.05, Supplementary table 1). We also performed a subgroup analysis according to the current use or nonuse of antihypertensive medication and found that the multivariable-adjusted change in systolic BP and the odds ratio for exercise-induced systolic BP elevation increased significantly with higher 3%ODI levels only in participants without antihypertensive medication (*p* for trend < 0.01), although there was no significant interaction (*p* for interaction = 0.09) (Supplemental table 2). Furthermore, when the highest quartile of systolic BP change was defined as exercise-induced systolic BP elevation, similarly significant associations were observed (*p* for trend < 0.05, Supplementary table 3).

## Discussion

In this cross-sectional study of an occupational Japanese population, systolic BP increased approximately 60 mmHg during exercise. Systolic BP values and odds ratios for exercise-induced systolic BP elevation increased significantly with higher 3%ODI levels after multivariate analysis. These findings suggest that there is a positive relationship between exercise-induced systolic BP elevation and 3%ODI levels, which may be partially explained by sympathetic hyperactivity. It would be clinically useful to rule out the possible coexistence of SRBD in patients with excessive increase in BP during exercise.

One previous controlled study of 115 middle-aged men (mean age: 52 years) with untreated hypertension also examined the difference in exercise-induced BP elevation between an OSA (mean AHI: 30 events/h) group and a non-OSA group [25]. In that study, systolic but not diastolic BP after exercise was significantly higher in the OSAS group than in the non-OSAS group (systolic BP: 198 ± 26 mmHg vs. 188 ± 24 mmHg, *p* < 0.03; diastolic BP: 92 ± 11 mmHg vs. 90 ± 10 mmHg, ns.), which is consistent with our present results.

Another previous cross-sectional study of 17 non-hypertensive young men (mean age: 35 years) examined the difference in exercise-induced BP elevation between OSA and non-OSA group [26]. In that study, diastolic but not systolic BP after exercise was significantly higher in the OSA group (mean AHI: 33 events/h) than that in the non-OSA group. (systolic BP: 215 ± 23 mmHg vs. 204 ± 26 mmHg, ns; diastolic BP: 115 ± 9 mmHg vs. 101 ± 8 mmHg, *p* < 0.01.) The results of this previous study differ from our present findings in that we observed, higher systolic but not higher diastolic BP after exercise in patients with SRBD. We speculate that age-related arterial stiffness may explain the different results in the BP responses to exercise (i.e., diastolic BP elevation in the young vs. systolic BP elevation in the middle-aged) between the young and middle-aged groups. The younger age of the participants (35 years in the previous study vs. 49 years in our study), which makes systolic BP less likely to increase, may be one of the possible reasons for the difference in results. The smaller sample size of the previous study, which would have made it more difficult to detect changes in systolic BP, could be another reason.

The present study showed that systolic BP during exercise increased significantly with higher 3%ODI levels, even after adjustment for several factors. One of the possible mechanisms underlying this result is that exercise-induced BP elevation may reflect sympathetic hyperactivity in patients with SRBD. In addition, higher 3%ODI levels were found to have a significant linear relationship with greater systolic BP elevation during exercise, which may further indicate that sympathetic activation due to hypoxia is responsible for the elevated BP. Of course, endothelial dysfunction [27, 28], arterial stiffness [27], renin-angiotensin-aldosterone (RAA) system activation [29], and cardiac dysfunction [30] also have been reported as factors contributing to exercise-induced BP elevation, in addition to sympathetic hyperactivity [31]. However, the finding that systolic BP rather than diastolic BP increased during exercise may support the idea that sympathetic hyperactivity was responsible.

Our present findings are clinically relevant from the following perspectives. SRBD may be present and could be related to excessive BP elevation when exercise-induced systolic BP elevation is observed. In support of this idea, it has been reported that sleep duration irregularity is associated with exercise-induced hypertension [32], and exercise-induced hypertension is known to be a risk factor for the development of hypertension and cardiovascular diseases [9], and chronic kidney disease [33]. Taking these issues into account, our findings could imply that SRBD patients are at higher risk for new onset of hypertension and cardiovascular diseases partly because of BP elevation during exercise. Encouraging patients to monitor their BP during physical activity could be beneficial. However, there are currently few effective strategies for managing individuals with excessive BP elevation during exercise. Our findings may also support the idea that identification of SRBD and therapeutic intervention such as CPAP therapy and mouthpiece use could help reduce the risk of future cardiovascular complications in individuals with excessive BP elevation during exercise. Future longitudinal studies are warranted to clarify the causal relationship between exercise-induced BP elevation and SRBD.

This study had several strengths. First, we enrolled a larger occupational population than similar previous studies. Second, this study included an occupational population of mostly male participants of younger middle age, without angina symptoms, which allowed us to examine the association between SRBD and exercise-induced BP elevation in fit men of younger middle age. Third, our results were based on reliable data with few missing values, because the participants were required to undergo regular health examinations, portable-device monitoring for overnight SpO2, and exercise ECG. Fourth, we evaluated not only post-exercise BP but also BP change during exercise, including the dose-response relationship according to 3%ODI levels.

However, several limitations of this study should be noted. First, portable device monitoring for overnight SpO2 was used to evaluate SRBD in the present study. Full polysomnography assesses apnea/hypopnea per hour by comprehensively recording biological activities during sleep. In contrast, the portable monitor for overnight SpO2 does not record sleep, which means that apnea/hypopnea per hour is calculated using the total recording time as the denominator, not the total sleep time. As a result, the AHI may have been underestimated, suggesting that the frequency of mild/moderate SRBD may also have been underestimated [17]. On the other hand, this does not affect the results of the dose-response relationship for the association between elevated 3%ODI and exercise-induced BP elevation. Second, since exercise ECG tests were conducted by different cardiologists at each of the 7 facilities, the time of sustained exercise at maximal intensity may have differed among the participants. This is, the participating cardiologists may have differed in regard to how many seconds of sustained exercise they required after reaching THR (i.e., whether to continue to the end of the stage). Longer duration of exercise at maximal exercise intensity may have resulted in greater exercise-induced BP elevation. Third, the effect of the class of antihypertensive agents on the association between the severity of SRBD and exercise-induced BP elevation was not investigated. The effect of antihypertensive medications (RAA system inhibitors or beta-blockers) on exercise-induced BP change remains controversial; since lack of data prevented this analysis here, these antihypertensive medications may have influenced the results in the present study. Systolic BP during exercise was not significantly elevated with higher 3%ODI levels among those taking antihypertensive medications in the present study, which may indicate that antihypertensive medication has a suppressive effect on exercise-induced BP elevation. Further studies that have larger sample sizes that include the data on the class of antihypertensive drugs are warranted. Fourth, the association between the severity of SRBD and BP recovery after exercise was not investigated. Nevertheless, the Framingham study reported that both exercise-induced BP elevation and delayed BP recovery were independent risk factors for hypertension [7], and thus these two phenomena might be attributed to common underlying mechanisms. Thus, the assessment of post-exercise BP may provide useful information to infer the presence of excessive exercise-induced BP elevation in clinical practice. Fifth, selection bias may have occurred because we recruited study participants from among train and bus drivers of a company based in Japan. In addition, the generalizability of the present findings to populations with different genetic backgrounds and lifestyles may be limited. Finally, potential residual confounding factors, such as underlying cardiovascular or pulmonary disease, were not evaluated. Since this was a cross-sectional study, the causal relationship between 3%ODI elevation and exercise-induced BP elevation remains to be elucidated.

### Perspective of Asia

Asians have been reported to have smaller jaws [34] and higher abdominal adiposity [35] than other ethnic groups, which may strengthen the association between 3%ODI and exercise-induced BP elevation. Further studies are warranted to elucidate the potential racial differences in the association between 3%ODI and exercise-induced BP elevation.

## Conclusions

Higher 3%ODI was significantly associated with greater exercise-induced BP elevation in this Japanese cross-sectional study. Our results suggest the potential presence of SRBD should be considered in individuals with exercise-induced systolic BP elevation.

## Supplementary information

Supplementary Table 1

Supplementary Table 2

Supplementary Table 3
