# Supplementary material for: Sleep-related breathing disorder in a Japanese occupational population and its association with exercise-induced blood pressure elevation
Source: Hypertens Res. 2024 Dec 5;48(2):754–62. doi: 10.1038/s41440-024-02050-6 (PMC11794129; doi:10.1038/s41440-024-02050-6)
Supplement: Supplementary file 1 — Supplementary Table 1 [file 41440_2024_2050_MOESM1_ESM.docx]

Supplementary Table 1.

Adjusted exercise-induced systolic BP change and the odds ratio for exercise-induced systolic BP elevation according to four 3%ODI levels

|  |  | number of subjects |  | age- and sex- adjusted |  | multivariate adjusted |
| --- | --- | --- | --- | --- | --- | --- |
| Exercise-induced systolic BP change |  |  |  |  |  |  |
| 0≤3%ODI<5 |  | 608 |  | 58.1±8.3 |  | 57.8±19.3 |
| 5≤3%ODI<10 |  | 217 |  | 60.1±8.4 |  | 58.7±19.5 |
| 10≤3%ODI<15 |  | 62 |  | 64.5±8.6 |  | 63.1±19.7 |
| 15≤3%ODI |  | 41 |  | 67.4±9.0 |  | 68.2±20.0 |
|  |  |  |  | p for trend<0.0001 |  | p for trend=0.01 |
|  |  | events/ total (%) |  | age- and sex- adjusted |  | multivariate adjusted |
| Odds ratio for  exercise-induced systolic BP elevation |  |  |  |  |  |  |
| 0≤3%ODI<5 |  | 280/608 (46.1%) |  | 1.00 (Reference) |  | 1.00 (Reference) |
| 5≤3%ODI<10 |  | 109/217 (50.2%) |  | 1.18 (0.86-1.61) |  | 1.13 (0.80-1.58) |
| 10≤3%ODI<15 |  | 41/62 (66.1%) |  | 2.19 (1.26-3.80) |  | 2.31 (1.27-4.21) |
| 15≤3%ODI |  | 27/41 (65.9%) |  | 2.32 (1.19-4.54) |  | 3.08 (1.46-6.47) |
|  |  |  |  | p for trend=0.005 |  | p for trend=0.002 |

Abbreviations: 3%ODI, 3% oxygen desaturation index; BP, blood pressure.

Data are presented as the adjusted mean values (standard error) or odds ratio (95% confidence interval).

Multi-variate analysis was performed by adjusting for age, sex, current alcohol drinking, current smoking, regular exercise, HbA1c, use of glucose-lowering agents, serum LDL cholesterol, serum HDL cholesterol, eGFR, BMI, systolic BP, heart rate in the annual medical examination, taking antihypertensive medication, and maximal exercise intensity at the time of exercise ECG.
