# Supplementary material for: Sleep-related breathing disorder in a Japanese occupational population and its association with exercise-induced blood pressure elevation
Source: Hypertens Res. 2024 Dec 5;48(2):754–62. doi: 10.1038/s41440-024-02050-6 (PMC11794129; doi:10.1038/s41440-024-02050-6)
Supplement: Supplementary file 2 — Supplementary Table 2 [file 41440_2024_2050_MOESM2_ESM.docx]

Supplementary Table 2.

Adjusted exercise-induced systolic BP change and the odds ratio for exercise-induced systolic BP elevation according to 3%ODI levels, stratified by current use or nonuse of antihypertensive medication

|  |  | number of subjects |  | age- and sex- adjusted |  | multivariate adjusted |
| --- | --- | --- | --- | --- | --- | --- |
| Exercise-induced systolic BP change |  |  |  |  |  |  |
| Antihypertensive medication: no |  |  |  |  |  |  |
| 0≤3%ODI<5 |  | 530 |  | 57.9±8.9 |  | 57.4±21.7 |
| 5≤3%ODI<15 |  | 227 |  | 60.3±9.1 |  | 58.8±22.0 |
| 15≤3%ODI |  | 30 |  | 70.0±10.0 |  | 71.0±22.5 |
|  |  |  |  | p for trend<0.0001 |  | p for trend=0.01 |
| Antihypertensive medication: yes |  |  |  |  |  |  |
| 0≤3%ODI<5 |  | 78 |  | 60.6±23.6 |  | 62.0±49.0 |
| 5≤3%ODI<15 |  | 52 |  | 63.3±23.1 |  | 61.6±49.0 |
| 15≤3%ODI |  | 11 |  | 58.3±23.5 |  | 54.2±50.0 |
|  |  |  |  | p for trend<0.0001 |  | p for trend=0.29 |
|  |  | events/ total (%) |  | age- and sex- adjusted |  | multivariate adjusted |
| Odds ratio  for exercise-induced systolic BP elevation |  |  |  |  |  |  |
| Antihypertensive medication: no |  |  |  |  |  |  |
| 0≤3%ODI<5 |  | 244/ 530 (46.0%) |  | 1.00 (Reference) |  | 1.00 (Reference) |
| 5≤3%ODI<15 |  | 118/ 227 (52.0%) |  | 1.26 (0.92-1.72) |  | 1.20 (0.85-1.69) |
| 15≤3%ODI |  | 22/ 30 (73.3%) |  | 3.38 (1.47-7.81) |  | 4.48 (1.78-11.30) |
|  |  |  |  | p for trend=0.01 |  | p for trend=0.006 |
| Antihypertensive medication: yes |  |  |  |  |  |  |
| 0≤3%ODI<5 |  | 36/ 78 (46.2%) |  | 1.00 (Reference) |  | 1.00 (Reference) |
| 5≤3%ODI<15 |  | 32/ 52 (61.5%) |  | 1.65 (0.80-3.42) |  | 1.68 (0.72-3.94) |
| 15≤3%ODI |  | 5/ 11 (45.5%) |  | 0.80 (0.22-2.90) |  | 0.93 (0.21-4.06) |
|  |  |  |  | p for trend=0.32 |  | p for trend=0.44 |
|  |  |  |  | p for interaction=0.11 |  | p for interaction=0.09 |

Abbreviations: 3%ODI, 3% oxygen desaturation index; BP, blood pressure.

Data are presented as the adjusted mean values (standard error) or odds ratio (95% confidence interval).

Multi-variate analysis was performed by adjusting for age, sex, current alcohol drinking, current smoking, regular exercise, HbA1c, use of glucose-lowering agents, serum LDL cholesterol, serum HDL cholesterol, eGFR, BMI, systolic BP, heart rate in the annual medical examination, and maximal exercise intensity at the time of exercise ECG.
