# Supplementary material for: Sleep-related breathing disorder in a Japanese occupational population and its association with exercise-induced blood pressure elevation
Source: Hypertens Res. 2024 Dec 5;48(2):754–62. doi: 10.1038/s41440-024-02050-6 (PMC11794129; doi:10.1038/s41440-024-02050-6)
Supplement: Supplementary file 3 — Supplementary Table 3 [file 41440_2024_2050_MOESM3_ESM.docx]

Supplementary Table 3.

Adjusted odds ratio for exercise-induced systolic BP elevation defined by the highest quartile according to 3%ODI levels, overall and stratified by current use or nonuse of antihypertensive medication

|  |  | events/ total (%) |  | age- and sex- adjusted |  | multi-adjusted (model 1) |
| --- | --- | --- | --- | --- | --- | --- |
| Odds ratio for exercise-induced systolic BP elevation |  |  |  |  |  |  |
| 0≤3%ODI<5 |  | 137/ 608 (22.5%) |  | 1 (Reference) |  | 1 (Reference) |
| 5≤3%ODI<15 |  | 82/ 279 (29.4%) |  | 1.44 (1.04-1.99) |  | 1.40 (0.98-2.01) |
| 15≤3%ODI |  | 15/ 41 (36.6%) |  | 2.03 (1.04-3.95) |  | 2.44 (1.18-5.07) |
|  |  |  |  | p for trend=0.02 |  | p for trend=0.02 |
|  |  | events/ total (%) |  | age- and sex- adjusted |  | multi-adjusted (model 2) |
| Odds ratio for exercise-induced systolic BP elevation |  |  |  |  |  |  |
| antihypertensive medication: no |  |  |  |  |  |  |
| 0≤3%ODI<5 |  | 116/ 530 (21.9%) |  | 1 (Reference) |  | 1 (Reference) |
| 5≤3%ODI<15 |  | 64/ 227 (28.2%) |  | 1.40 (0.98-2.00) |  | 1.39 (0.93-2.07) |
| 15≤3%ODI |  | 12/ 30 (40.0%) |  | 2.43 (1.14-5.22) |  | 2.94 (1.27-6.79) |
|  |  |  |  | p for trend=0.02 |  | p for trend=0.03 |
| antihypertensive medication: yes |  |  |  |  |  |  |
| 0≤3%ODI<5 |  | 21/ 78 (26.9%) |  | 1 (Reference) |  | 1 (Reference) |
| 5≤3%ODI<15 |  | 18/ 52 (34.6%) |  | 1.24 (0.57-2.71) |  | 1.01 (0.38-2.70) |
| 15≤3%ODI |  | 3/ 11 (27.3%) |  | 0.80 (0.19-3.40) |  | 0.68 (0.13-3.63) |
|  |  |  |  | p for trend=0.79 |  | p for trend=0.89 |
|  |  |  |  | p for interaction=0.48 |  | p for interaction=0.42 |

Abbreviations: 3%ODI, 3% oxygen desaturation index; BP, blood pressure.

Data are presented as the adjusted mean values (standard error) or odds ratio (95% confidence interval).

Multi-variate analysis (model 1) was performed by adjusting for age, sex, current alcohol drinking, current smoking, regular exercise, HbA1c, use of glucose-lowering agents, serum LDL cholesterol, serum HDL cholesterol, eGFR, BMI, systolic BP, heart rate in the annual medical examination, antihypertensive medication use, and maximal exercise intensity at the time of exercise ECG.

Multi-variate analysis (model 2) was performed by adjusting for all variables in model 1 except for antihypertensive medication use.
